# Inter-Limb Asymmetry and Its Limited Role in Physical Performance and Match Demands in Football Players with Spastic Hemiparesis—An Exploratory Team Study

**DOI:** 10.3390/jfmk11030276

**Published:** 2026-07-17

**Authors:** Iván Peña-González, Alba Roldán, Bartolomé Leal Barquero, Alejandro Caña-Pino, Manuel Moya-Ramón

**Affiliations:** 1 Sports Research Centre Institute, Department of Sports Sciences, Miguel Hernández University of Elche, 03202 Elche, Spain; ipena@umh.es (I.P.-G.); aroldan@umh.es (A.R.); mmoya@umh.es (M.M.-R.); 2 Spanish Federation of Sports for people with CP and ABI (FEDPC), 28008 Madrid, Spain; bartolome.leal@mutualidaddefutbolistas.com; 3 Surgical Medical-Therapy Department, Medicine Faculty and Health Sciences, University of Extremadura, 06006 Badajoz, Spain; 4 Research Group PhysioH (Fisioterapia e Hipoterapia), University of Extremadura, 06006 Badajoz, Spain

**Keywords:** cerebral palsy, spastic hemiparesis, inter-limb asymmetry, isometric strength, football, physical performance, match load, plantar flexors

## Abstract

**Background:** Inter-limb asymmetry has been widely studied as a potential determinant of physical performance in able-bodied athletes; however, its functional relevance in athletes with neurological impairments such as spastic hemiparesis remains unclear. This study aimed to examine the associations between lower-limb isometric strength, inter-limb asymmetry, physical performance, and match external-load variables in elite CP football players. **Methods**: Eleven male football players with spastic hemiparesis from the Spanish national team competing at the 2024 IFCPF World Cup participated in this observational cross-sectional study. Maximal isometric strength of the plantar flexors, adductors, and hamstrings was assessed using a belt-stabilised dynamometer. Inter-limb asymmetry was calculated as a percentage difference between affected and non-affected limbs. Physical performance was evaluated using sprint, change-of-direction, dribbling, and intermittent endurance tests. Match external-load variables were collected during official competition using inertial measurement units. Associations were analysed using Spearman’s rank correlations, and between-group comparisons were conducted using a median split based on asymmetry magnitude. **Results:** Inter-limb asymmetry did not significantly differentiate physical performance outcomes across any field-based tests (*p* > 0.05). Associations between isometric strength or asymmetry and field-based performance were limited and did not remain statistically significant after false discovery rate correction. In contrast, plantar flexor asymmetry showed significant negative associations with mechanical work (ρ = −0.84; q = 0.010) and metabolic power (ρ = −0.83; q = 0.010), which remained robust after multiple-comparison control. **Conclusions**: Inter-limb strength asymmetry did not appear to be a primary determinant of field-based physical performance in CP football players with spastic hemiparesis. Most associations between strength, asymmetry, and performance should be considered exploratory. However, plantar flexor asymmetry showed a consistent association with selected mechanical and metabolic match-load variables, suggesting that neuromuscular asymmetry may influence specific aspects of match demands. Given the exploratory team-study design and limited sample size, these findings should be interpreted cautiously and require confirmation in larger cohorts.

## 1. Introduction

Cerebral palsy (CP) is the most prevalent childhood-onset physical disability and encompasses a range of permanent motor impairments, including spasticity, muscle weakness, impaired selective motor control, and altered intersegmental coordination [1,2]. Spastic hemiparesis, characterised by unilateral involvement of the upper and lower extremities, is one of the most common CP subtypes [1]. The unilateral neuromuscular involvement associated with this condition frequently results in marked differences in force production and motor control between the affected and non-affected limbs [3,4].

CP football is a 7-a-side para-sport for athletes with CP or acquired brain injury and is characterised by repeated high-intensity actions, including sprints, changes in direction, accelerations, and decelerations, interspersed with lower-intensity activity [4,5,6]. Players are classified into three sport classes (FT1, FT2, and FT3) according to the impact of their impairment on football-specific activity, with FT1 representing the greatest and FT3 the lowest activity limitation [4]. Sport class and impairment characteristics substantially influence physical performance and match demands, with less impaired players generally achieving greater high-intensity running and performing more explosive neuromuscular actions [4,5,6,7,8,9].

Within this sport-specific context, lower-limb strength and inter-limb asymmetry may be relevant to understanding individual differences in physical performance and match external load. Inter-limb asymmetry has been widely examined in able-bodied athletes, although its associations with sprint and change-of-direction (COD) performance are task-dependent and inconsistent [5,6,7]. In athletes with spastic hemiparesis, however, asymmetry may represent a more persistent characteristic of the neuromuscular profile rather than a transient training-related imbalance. Despite the marked unilateral impairments observed in CP football players, the extent to which lower-limb strength and inter-limb asymmetry are associated with football-specific performance and competitive match demands remains unclear. Understanding these relationships may provide relevant information for neuromuscular profiling and the interpretation of match-load data in CP football. However, evidence in elite players remains scarce, partly because of the limited availability of homogeneous samples competing at the highest international level.

Previous evidence suggests that lower-limb neuromuscular characteristics may be relevant to functional performance in individuals with CP. Plantar flexor isometric strength has been shown to explain a substantial proportion of the variance in functional performance in adults with CP [10], while lower-limb spasticity in CP football players has shown negative associations with balance, horizontal jump capacity, and COD performance [11]. However, the functional relevance of inter-limb asymmetry remains unclear. Recent evidence in international CP footballers with spastic hemiparesis found no significant associations between anthropometric asymmetry indices and physical performance, whereas morphological characteristics of the non-affected limb were associated with strength, jump, and sprint outcomes [12]. These findings suggest that the contribution of strength and asymmetry to sport-specific performance in athletes with unilateral neurological impairment may be complex and task-dependent.

Despite these advances, the relationship between lower-limb isometric strength, inter-limb asymmetry, and sport-specific performance in CP football remains poorly characterised. In particular, it is unclear whether strength and asymmetry across specific lower-limb muscle groups are associated with field-based physical performance or with external-load demands during official competition. Furthermore, the extent to which different magnitudes of inter-limb asymmetry differentiate physical performance outcomes in players with spastic hemiparesis remains unknown. Examining these relationships may contribute to a better understanding of how individual neuromuscular profiles interact with the physical demands of elite CP football and may inform player monitoring and training prescription.

Among the lower-limb muscle groups, the plantar flexors, hamstrings, and hip adductors are commonly analysed because of their key contribution to sprint propulsion, deceleration, change-of-direction mechanics, and frontal-plane stability during football-specific actions. In athletes with spastic hemiparesis, impairments affecting these muscle groups may therefore have important functional implications for both field-based performance and match physical demands. Therefore, this exploratory team study aimed to examine the associations between lower-limb isometric strength, inter-limb asymmetry, field-based physical performance, and match external-load variables in football players with spastic hemiparesis from a national team competing at the 2024 IFCPF World Cup. It was hypothesised that strength of the plantar flexors, hamstrings, and hip adductors, together with inter-limb asymmetry, would be associated with field-based physical performance and match external-load variables, although the magnitude and direction of these associations were expected to vary according to the specific task and performance domain examined. Given the exploratory nature of the study and the highly specific elite para-sport sample, the findings were intended to identify potential patterns of association and generate hypotheses for future research rather than provide confirmatory population-level evidence.

## 2. Materials and Methods

### 2.1. Design

A cross-sectional observational study was conducted. Given the highly specific elite para-sport population and the inclusion of players from a single national team, the investigation was conceived as an exploratory team study aimed at identifying potential patterns of association rather than providing confirmatory population-level estimates. Isometric strength and field-based physical performance assessments were conducted during the training period immediately prior to the 2024 IFCPF World Cup, ensuring a minimum of 24 h of rest before testing to minimise the potential influence of fatigue on performance outcomes. Match external-load data were collected during official matches played in the same competition.

### 2.2. Participants

Eleven male football players with spastic hemiparesis from the Spanish national CP football team participated in the study. According to the IFCPF classification system, the sample comprised 11 male outfield players with spastic hemiparesis (FT1, n = 2; FT2, n = 7; FT3, n = 2). According to their primary playing position during the competition, players were classified as defenders (n = 5), midfielders (n = 3), and forwards (n = 3). Participants had a mean age of 23.73 ± 4.34 years, body mass of 74.68 ± 9.35 kg, and height of 178.42 ± 8.32 cm. All players held a valid competitive licence issued by the Spanish Federation for Athletes with Cerebral Palsy and had at least three years of international competitive experience. Inclusion criteria required players to be injury-free for at least 12 months prior to data collection. All participants provided written informed consent in accordance with the Declaration of Helsinki. The study protocol was approved by the Research Ethics Committee of Miguel Hernández University of Elche (approval number: 240426200237; Reference: ADH.DES.IPG.JFM.24.), and all participants provided written informed consent before participation.

### 2.3. Procedures

#### 2.3.1. Isometric Strength and Asymmetry Assessment

Maximal isometric strength was assessed using a portable load-cell dynamometer (K-Pull V3, Kinvent^®^, Montpellier, France), which enables the quantification of tensile force through a belt-stabilised setup. The device has a maximum capacity of 300 kg, with a sensitivity of 500 g and a measurement precision of 0.1% of the recorded value. Force data were transmitted via Bluetooth to a dedicated mobile application and recorded in Newtons (N). Force signals were sampled and processed via the manufacturer’s proprietary software. Portable Kinvent dynamometers have demonstrated good-to-excellent intra- and inter-rater reliability for the assessment of muscle strength across upper- and lower-limb muscle groups [13]. Furthermore, all dynamometry assessments were conducted by a licensed physiotherapist with more than 10 years of clinical and sports-performance experience. To minimise potential evaluator bias, strength assessments and field-based physical performance tests were performed by different evaluators.

All measurements were performed unilaterally on both affected and non-affected limbs using a belt-stabilised configuration, whereby the dynamometer was connected to the participant via a distal strap and anchored to the examiner through a rigid adjustable belt. This configuration was used to minimise examiner-related variability and ensure consistent force direction across trials [14].

Participants completed a standardised warm-up consisting of 5 min of low-intensity running, followed by dynamic mobility exercises and two to three submaximal familiarisation contractions for each muscle group.

Three muscle groups were evaluated: hamstrings, plantar flexors, and hip adductors. To facilitate familiarisation with the testing procedure and maximise the validity of maximal efforts, all participants were first assessed on the non-affected limb before testing the affected limb. This approach is consistent with recommendations in neuromuscular testing protocols, where familiarisation is known to influence maximal force output [14,15,16]. All muscle groups were assessed in a fixed order (hamstrings, plantar flexors, and hip adductors) to ensure consistency across participants and minimise variability associated with randomised testing sequences. Although randomisation of test order may reduce systematic fatigue effects, a fixed sequence was used due to the exploratory nature of the study and the need for protocol standardisation [14,15,16]. Participants were instructed to maintain a stable body position during all contractions, and visual observation by the examiner ensured that no compensatory movements occurred at the trunk or proximal joints.

Hamstring strength was assessed with participants in a prone position, with the knee flexed to 90°, while the dynamometer strap was secured just proximal to the ankle joint. Participants were instructed to perform a maximal isometric knee flexion against the fixed resistance.

Plantar flexor strength was assessed in a seated position with the knee extended and the ankle in a neutral position. The dynamometer was attached to the forefoot via a strap, and participants exerted maximal plantar flexion force against the fixed resistance. Although this position does not isolate a specific plantar flexor muscle and involves substantial gastrocnemius contribution, it was used as a functional assessment of plantar flexor strength.

**Figure 1:**
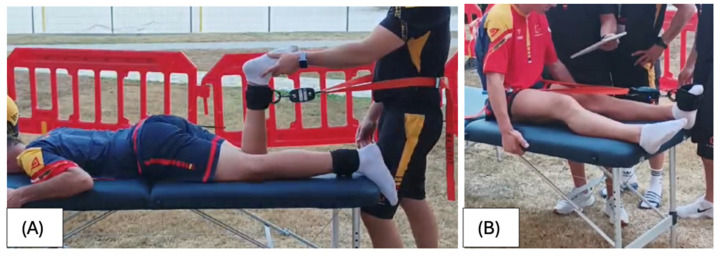
Standardised setup for isometric strength assessment using a belt-stabilised dynamometry system (K-Pull V3, Kinvent^®^). (**A**) Hamstring assessment performed in prone position with the knee flexed at 90°. (**B**) Plantar flexor assessment performed in a seated position with the knee extended. Hip adductor strength was assessed using a similar belt-stabilised configuration in a supine position, with the limb placed in slight abduction and force applied medially against external fixation. In all conditions, the dynamometer was attached distally to the limb and anchored to the examiner using a rigid belt to ensure a consistent direction of force application and to minimise measurement variability.

Hip adductor strength was assessed in a supine position, with the tested limb positioned in slight abduction. The dynamometer was attached distally to the limb, and participants were instructed to perform a maximal isometric adduction against the resistance provided via the belt-stabilised system (Figure 1).

For each muscle group and limb, participants performed three maximal voluntary isometric contractions (MVICs) of 5 s duration, with a 10 s rest interval between trials [14,15,16]. Strong verbal encouragement was provided during all contractions to maximise effort. The highest force value (peak force, N) obtained across the three trials was retained for analysis. Peak force (N) was used as the primary outcome variable for all analyses. This approach is consistent with established practice in isometric strength assessment, where peak force demonstrates high intra-session reliability and provides a valid representation of maximal neuromuscular capacity [16,17].

Inter-limb asymmetry was calculated for each muscle group using the following equation:

Asymmetry (%) = (|Non-affected − Affected|/max[Non-affected, Affected]) × 100

The non-affected limb was operationally considered the stronger reference limb for asymmetry calculations. This method has been widely adopted in sports science research for quantifying inter-limb differences, as it accounts for the magnitude of the strongest limb and reduces bias associated with directional dominance [18].

#### 2.3.2. Field-Based Physical Performance Assessment

Physical performance was evaluated using a battery of field tests, including a 30 m linear sprint test (with split times at 5, 15, and 30 m to assess acceleration and maximal velocity), the Modified Agility Test (MAT) to evaluate change-of-direction ability, a ball-dribbling version of the MAT (MAT-B) to assess dribbling capacity, and the Yo-Yo Intermittent Recovery Test Level 1 (Yo-Yo IR1) to evaluate intermittent endurance. Sprint, change-of-direction, and dribbling performances were measured using photocell timing gates (Witty System, Microgate, Bolzano, Italy). Two trials were performed for each test, with 3 min of passive recovery between attempts, and the best performance was retained for analysis. Players started voluntarily from a standing position, located 30 cm behind the first timing gate. Dribbling ability was calculated as the difference (in seconds) between MAT-B and MAT performance, thereby isolating the additional time required to complete the task with ball control beyond change-of-direction ability alone. The Yo-Yo IR1 test was performed once, and total distance covered (m) was recorded.

All tests were conducted on the same day, in a fixed order progressing from shorter to longer duration tasks to minimise fatigue effects. Participants wore their usual football boots and the same standardised ball was used for all participants during the MAT-B. All field-based physical performance assessments were conducted on the same outdoor artificial-grass football pitch under comparable environmental conditions and players were instructed to perform maximally in all tests. Players were already familiar with the testing procedures, as these assessments were routinely used as part of the team’s physical performance monitoring.

#### 2.3.3. Match External-Load Variables

Match external-load data were collected using inertial measurement units (WIMU Pro™, RealTrack Systems, Almería, Spain), sampling at 18 Hz. These devices have demonstrated validity and reliability for assessing external load in team sports [19]. Players wore the devices during all official matches, positioned in a specific harness located between the scapulae. Match external-load data were obtained from six official matches played during the 2024 IFCPF World Cup, resulting in a total of 54 player-match observations. Goalkeepers were excluded from the analysis. Only player-match observations with a minimum individual playing time of 10 min were included. All time-dependent external-load variables were subsequently normalised to individual playing time and expressed per minute.

To enhance interpretability and reduce redundancy, variables were grouped into functional domains:

- Global locomotor load: total distance per minute (m·min^−1^) and average velocity (km·h^−1^), representing overall movement volume and pace
- Locomotor intensity distribution: distance per minute at low, moderate, and high intensities (m·min^−1^), reflecting distribution of movement across speed zones
- Neuromuscular actions: number of accelerations and decelerations per minute (n·min^−1^), maximum and average acceleration/deceleration (m·s^−2^), and distance and frequency of moderate- and high-intensity accelerations/decelerations
- Explosive and sprint actions: explosive distance (m·min^−1^), sprint frequency (n·min^−1^), sprint distance (m·min^−1^), and maximum velocity (km·h^−1^)
- Mechanical and metabolic load: Player Load (AU·min^−1^), mechanical work (kJ·min^−1^), metabolic power (W·kg^−1^), and high metabolic load distance (m·min^−1^), providing an integrated estimate of the mechanical and energetic demands of match play.

Speed zones were defined as follows: low-intensity distance, 6–12 km·h^−1^; moderate-intensity distance, >12–18 km·h^−1^; high-intensity distance, >18 km·h^−1^; and sprint distance, >24 km·h^−1^. Acceleration and deceleration thresholds were defined as moderate intensity between 2 and 3 m·s^−2^ and high intensity >3 m·s^−2^, with the corresponding negative thresholds applied for decelerations (<−2 to −3 m·s^−2^ and <−3 m·s^−2^, respectively). Explosive distance, metabolic power, and high metabolic load distance were calculated by the WIMU Pro™ software (version 2.0.0.1) according to the manufacturer’s proprietary algorithms and predefined thresholds.

### 2.4. Statistical Analysis

Descriptive statistics are reported as mean ± standard deviation. Normality of the data was assessed using the Shapiro–Wilk test. Given the small sample size and the non-normal distribution of several variables, non-parametric analyses were employed. For the correlation analyses, match external-load variables were averaged across all available matches for each player. Consequently, all correlation analyses were performed using one aggregated value per participant (n = 11), rather than individual player-match observations. Associations between isometric strength, inter-limb asymmetry, physical performance, and match external-load variables were examined using Spearman’s rank correlation coefficients (ρ). Given the exploratory nature of the study and the number of correlations performed, the Benjamini–Hochberg false discovery rate (FDR) procedure was applied as a sensitivity analysis to control for multiple comparisons. FDR correction was applied across the complete family of strength/asymmetry–field performance correlations and within a priori functional domains for match external-load variables. Raw *p*-values were retained for exploratory purposes, whereas FDR-adjusted *p*-values (q-values) were used to assess the robustness of significant associations. Associations with q < 0.05 were considered robust, while those significant only at the unadjusted level were interpreted as exploratory. Paired comparisons between affected and non-affected limb isometric strength values were performed using the Wilcoxon signed-rank test. To explore the potential impact of asymmetry magnitude, players were categorised into lower- and higher-asymmetry groups for each muscle group using a median split. This approach was adopted to facilitate group comparisons while preserving statistical power given the limited sample size. Between-group comparisons in physical performance variables were conducted using the Mann–Whitney U test. Effect sizes were calculated using rank-biserial correlation (r). Statistical significance was set at *p* < 0.05 for unadjusted analyses and q < 0.05 for FDR-adjusted analyses. Primary statistical analyses were performed using JASP software (version 0.19.1.0), and FDR-adjusted *p*-values were calculated using the Benjamini–Hochberg procedure.

## 3. Results

In linear sprint assessments, players recorded times of 1.19 ± 0.07 s (5 m), 2.80 ± 0.20 s (15 m), and 4.83 ± 0.29 s (30 m). Performance in the MAT was 5.90 ± 0.36 s, increasing to 8.68 ± 0.78 s in the ball-dribbling version (MAT-B). Dribbling ability, calculated as the difference between MAT-B and MAT, was 2.78 ± 0.64 s. Intermittent endurance, assessed using the Yo-Yo IR1, resulted in a mean distance of 1163.64 ± 416.25 m. Isometric strength values and inter-limb asymmetry indices are reported in Table 1. Across all muscle groups, the affected limb produced significantly lower isometric force values than the non-affected limb (all *p* < 0.001). The greatest asymmetry was observed in the hamstrings (81.81 ± 40.30%), followed by the adductors (49.83 ± 39.17%) and the plantar flexors (46.58 ± 42.47%).

Spearman’s correlation coefficients between isometric strength, asymmetry, and physical performance variables are presented in Table 2. In general, isometric strength of both limbs tended to be negatively associated with sprint times and agility performance, indicating that higher strength values were generally related to better performance. At the unadjusted level, a positive correlation was observed between non-affected adductor strength and Yo-Yo IR1 performance (ρ = 0.63; *p* = 0.038). Similarly, at the unadjusted level, greater plantar flexor asymmetry was associated with faster MAT-B performance (i.e., lower completion times; ρ = −0.64; *p* = 0.035). However, neither association remained statistically significant after FDR correction across the complete family of correlations (both q = 0.699). Therefore, these associations should be considered exploratory rather than robust findings.

Spearman’s correlation coefficients between inter-limb asymmetry and match external-load variables, grouped by the predefined functional domains, are reported in Table 3. No significant associations were observed for adductor or hamstring asymmetry across any external-load variable. Plantar flexor asymmetry showed a pattern of moderate associations with selected neuromuscular variables, including accelerations and decelerations per minute (ρ = 0.54), distance covered during high-intensity accelerations (ρ = 0.55), and maximum velocity (ρ = 0.54); however, none of these associations reached statistical significance. Within the mechanical and metabolic load domain, plantar flexor asymmetry was negatively associated at the unadjusted level with Player Load per minute (ρ = −0.62; *p* = 0.040), mechanical work per minute (ρ = −0.84; *p* = 0.001), and metabolic power (ρ = −0.83; *p* = 0.002). After FDR correction within the predefined mechanical and metabolic load domain, the associations between plantar flexor asymmetry and mechanical work per minute (ρ = −0.84; 95% CI [−0.97, −0.44]; q = 0.010) and metabolic power (ρ = −0.83; 95% CI [−0.97, −0.42]; q = 0.010) remained statistically significant, whereas the association with Player Load did not (q = 0.161). Although these confidence intervals support a negative direction of association, their width reflects substantial uncertainty around the precise magnitude of the relationships. In line with this pattern, the moderate-asymmetry group showed higher values than the higher-asymmetry group for metabolic power (97.65 ± 38.83 vs. 53.13 ± 4.90 W·kg^−1^; U = 29.00; *p* = 0.009; r = 0.93) and mechanical work per minute (1.85 ± 0.77 vs. 1.01 ± 0.10 kJ·min^−1^; U = 29.00; *p* = 0.009; r = 0.93). Although acceleration and deceleration frequencies showed identical Spearman correlation coefficients with the three asymmetry measures, the underlying acceleration and deceleration values were not identical. The matching coefficients resulted from the highly similar rank ordering of players across both variables.

Between-group comparisons based on asymmetry magnitude (median split) are presented in Table 4. No statistically significant differences were observed between moderate- and higher-asymmetry groups in any physical performance variable, including sprint performance (5–30 m), change-of-direction ability (MAT and MAT-B), dribbling ability, and Yo-Yo IR1 performance (all *p* > 0.05). However, effect size analysis revealed small-to-moderate differences in some variables. Notably, a moderate effect size was observed for MAT-B performance in relation to plantar flexor asymmetry (r = 0.47). Overall, these results indicate that inter-limb strength asymmetry was not associated with meaningful differences in physical performance in this sample.

## 4. Discussion

The aim of this study was to examine the relationships between lower-limb isometric strength, inter-limb asymmetry, physical performance, and match external-load variables in football players with spastic hemiparesis. Given the case-study nature of this investigation, involving a small sample of players from a single international-level team, the findings should be interpreted within this specific context. The main results indicated that (i) inter-limb asymmetry did not meaningfully differentiate physical performance outcomes, (ii) associations between isometric strength or asymmetry and field-based performance were limited and did not remain significant after controlling the false discovery rate, and (iii) plantar flexor asymmetry showed a more consistent pattern of association with mechanical and metabolic match-load variables, with the associations involving mechanical work and metabolic power remaining significant after FDR correction. Given the exploratory team-study design and the limited sample available within this highly specific international competitive context, the findings should primarily be interpreted as patterns of association rather than as confirmatory evidence of causal relationships.

From a performance perspective, the absence of significant between-group differences across all asymmetry classifications suggests that inter-limb strength asymmetry is not a primary determinant of sprinting, change-of-direction ability, dribbling performance, or intermittent endurance in this population. This is broadly consistent with a growing body of evidence challenging the assumption that asymmetry is inherently detrimental to performance. In able-bodied athletes, the asymmetry–performance relationship has proven highly inconsistent, varying substantially depending on the physical quality assessed, the task structure, and the methodology employed [5,6]. For instance, Exell et al. [20] found no association between inter-limb asymmetry and sprint performance in trained sprinters, while previous reviews have shown that inter-limb asymmetries are highly prevalent in athletic populations and do not necessarily impair physical performance, suggesting that asymmetry may represent a common functional characteristic rather than a deviation from optimal motor organisation [5,6]. In this regard, commonly used asymmetry thresholds (e.g., 10–15%) have been increasingly questioned given their limited empirical basis and poor cross-test agreement [21,22], supporting the need for an individualised rather than threshold-based approach to asymmetry interpretation.

In athletes with neurological impairments such as spastic hemiparesis, this argument gains additional weight. Asymmetry in this population is not a modifiable training variable but an intrinsic and structurally stable feature of the condition, arising from spasticity, reduced selective motor control, and muscle weakness differentially affecting the two limbs [2,3]. In the present study, the magnitude of inter-limb asymmetry was notably high across all muscle groups, particularly in the hamstrings. This finding is consistent with the neuromuscular profile of spastic hemiparesis, where unilateral impairments lead to marked strength deficits in the affected limb. Interestingly, asymmetry magnitude differed across muscle groups, with the hamstrings exhibiting the greatest inter-limb deficits. This may reflect the greater susceptibility of biarticular muscles to neuromuscular impairment in spastic hemiparesis, as well as their involvement in complex motor functions such as knee flexion during locomotion. Furthermore, the non-affected limb produced significantly higher isometric force values across all muscle groups, confirming marked unilateral strength deficits consistent with the neuromuscular profile of spastic hemiparesis. This strength imbalance may contribute to the compensatory motor strategies commonly observed in athletes with unilateral neurological impairments. Despite these substantial asymmetry magnitudes, their limited association with physical-performance outcomes supports the notion that asymmetry in this population represents a structural characteristic rather than a direct performance-limiting factor. Similarly, the unadjusted association between non-affected adductor strength and intermittent endurance did not survive FDR correction. Although the potential contribution of the non-affected limb to compensatory force production remains of interest in athletes with unilateral impairment, the present association should be considered hypothesis-generating and requires confirmation in larger samples.

Consistent with this, inter-limb asymmetries in jump performance have been previously quantified in international CP footballers across sport classes, with greater magnitudes in FT1 and FT2 players, yet without significant inter-limb differences in COD performance [11,23,24]. Similarly, recent evidence in international CP footballers with spastic hemiparesis suggests that substantial inter-limb asymmetries do not necessarily translate into impaired sport-specific performance. Maggiolo et al. [12] reported no significant associations between anthropometric asymmetry indices and sprinting, jumping, or agility performance, while performance was more strongly related to the morphological characteristics of the non-affected limb. Furthermore, previous work in CP football has highlighted the influence of impairment-related factors, such as spasticity, on motor performance outcomes [11], supporting the notion that athletes may adopt compensatory strategies to maintain functional performance despite marked neuromuscular asymmetries. Taken together, these findings converge on the idea that athletes with spastic hemiparesis may rely on compensatory mechanisms centred on the non-affected side to sustain performance across a range of physical tasks, effectively decoupling asymmetry magnitude from performance outcomes [11,12,24,25].

The apparent dissociation between asymmetry and performance may also be explained by the biomechanical demands of the tasks themselves. Sprint and COD tests, unlike isolated unilateral assessments, permit considerable flexibility in motor strategy, allowing athletes to redistribute mechanical demands across limbs and adopt alternative coordination patterns. This has been observed in able-bodied populations: Dos’Santos et al. [26] reported that inter-limb asymmetries in unilateral tasks were not necessarily detrimental to COD performance, suggesting that the coordinative and neuromuscular demands of the two task types diverge substantially. These considerations reinforce the need to avoid over-interpreting asymmetry indices derived from isolated strength tests as universal predictors of sport-specific performance.

At the unadjusted level, greater plantar flexor asymmetry was associated with faster MAT-B performance, as indicated by the negative correlation with completion time. However, this association did not remain statistically significant after FDR correction and should therefore be regarded as an exploratory finding rather than a robust association. The unexpected direction of this relationship suggests that greater asymmetry does not necessarily translate into poorer performance in technically complex tasks and may instead reflect individual compensatory motor strategies developed by elite CP football players. Given the small sample size and the absence of similar associations with MAT or other field-based performance variables, no firm functional interpretation can be drawn. Nevertheless, the greater task complexity and ecological validity of the MAT-B warrant further investigation, as integrating locomotion with ball control may reveal movement strategies that are not captured by standard change-of-direction tests. Future studies should determine whether this pattern reflects genuine compensatory adaptations or simply sampling variability.

With respect to match external-load variables, the findings reinforce the importance of contextual interpretation. Adductor and hamstring asymmetry showed no meaningful associations with any match-load domain, whereas plantar flexor asymmetry displayed a more consistent pattern of association with selected match-load variables. Importantly, after controlling the false discovery rate within the predefined functional domains, only the associations between plantar flexor asymmetry and mechanical work per minute (ρ = −0.84; q = 0.010) and metabolic power (ρ = −0.83; q = 0.010) remained statistically significant. Despite the magnitude of these correlation coefficients, their confidence intervals reflected substantial uncertainty around the estimated strength of the associations, as expected given the limited sample size. Therefore, the large observed coefficients should not be interpreted as precise estimates of the underlying population relationships. Rather, they indicate a potentially relevant pattern of association within this specific national-team sample that requires replication in larger cohorts. In contrast, the unadjusted association with Player Load and the moderate correlations observed with selected neuromuscular variables did not survive multiple-comparison control. Therefore, the present findings do not support a broad association between plantar flexor asymmetry and overall match load; rather, they identify a specific exploratory pattern within the mechanical and metabolic load domain. Between-group comparisons were consistent with this pattern, as players with moderate plantar flexor asymmetry accumulated greater metabolic power and mechanical work per minute during matches than their higher-asymmetry counterparts.

These findings should not be interpreted as evidence that greater plantar flexor asymmetry directly limits a player’s physical capacity or causes lower mechanical or metabolic match loads. Mechanical work and metabolic power are derived composite metrics obtained from locomotor and acceleration-related data and are therefore not statistically or physiologically independent. Consequently, the similar direction and magnitude of their associations with plantar flexor asymmetry may reflect shared underlying information rather than independent effects across multiple dimensions of match load. In this regard, the persistence of both associations after FDR correction should be interpreted as a consistent pattern within a single functional domain rather than as two independent pieces of evidence supporting a causal role of plantar flexor asymmetry.

Furthermore, although external-load variables dependent on playing time were normalised relative to individual playing time and expressed per minute, this procedure does not eliminate the potential influence of match-contextual and player-related factors. Match demands in CP football are strongly influenced by sport class, playing position, tactical role, match context, and individual competitive exposure. Given the sport-class distribution of the present sample (2 FT1, 7 FT2, and 2 FT3 players), classification-related differences in activity limitation may partly contribute to the observed associations. Similarly, positional and tactical roles may determine players’ involvement in high-intensity and mechanically demanding actions. Therefore, the observed mechanical and metabolic load pattern may reflect differences in competitive exposure or movement engagement associated with individual functional and contextual profiles rather than a direct biomechanical consequence of plantar flexor asymmetry [4,8,27].

From a biomechanical perspective, plantar flexor function remains a plausible factor of interest because of its contribution to propulsion, ankle stabilisation, and energy return during locomotion. However, the present exploratory data cannot establish whether plantar flexor asymmetry independently contributes to the observed match-load pattern. The associations identified here should therefore be regarded as hypothesis-generating and examined in larger, multi-team samples using multivariable approaches capable of accounting for positional role, sport class, playing exposure, and other relevant contextual covariates.

An additional explanation for the apparent discrepancy between the absence of robust associations with short field-based tests and the relationships observed with mechanical work and metabolic power during match play lies in the different physiological and biomechanical demands of these assessments. Sprint and change-of-direction tests assess brief maximal efforts performed under controlled conditions, whereas official matches require repeated multidirectional actions, prolonged locomotor activity, and continuous adaptation to tactical and environmental constraints. Under these sustained demands, athletes with spastic hemiparesis may rely more heavily on compensatory movement strategies, potentially increasing the functional relevance of plantar flexor asymmetry during match play [4,6,11]. Nevertheless, this interpretation remains speculative, as fatigue, movement strategies, and biomechanical compensations were not directly assessed in the present study. Future investigations combining biomechanical analyses with match-performance monitoring are warranted to determine whether cumulative neuromuscular demands contribute to the associations observed in competition [4,27].

Although not a primary focus of this study, the pronounced strength deficits and asymmetry magnitudes observed—particularly in the hamstrings (81.81 ± 40.30%)—are noteworthy from a potential injury risk perspective. In able-bodied football, inter-limb asymmetries in knee flexor and extensor strength have been associated with elevated risk of muscle injury and reinjury [28], although large systematic reviews have reported mixed and generally low-to-moderate quality evidence for a generalizable asymmetry–injury relationship [7,29]. In CP footballers, unilateral motor impairments commonly result in substantial strength differences between the affected and non-affected limbs, which may contribute to asymmetrical loading patterns during sport-specific activities [11].

Although these asymmetries may theoretically contribute to altered loading patterns resulting from co-contraction and impaired selective motor control, the present study did not assess injury incidence and therefore cannot establish any relationship between asymmetry and potential injury risk. Therefore, the high hamstring asymmetry observed in the present sample should be interpreted as a neuromuscular characteristic that may warrant monitoring in elite CP football players. However, any potential implication for injury risk remains speculative and requires confirmation through longitudinal studies specifically designed to examine injury occurrence.

### 4.1. Methodological Considerations and Limitations

Several methodological considerations and limitations should be acknowledged. First, the small sample size (n = 11) limits statistical power, the precision of correlation estimates, and the generalisability of the findings. However, the sample comprised the complete group of male players with spastic hemiparesis from a single national team competing at the 2024 IFCPF World Cup, reflecting the inherent recruitment constraints associated with research in highly specific elite para-sport populations. Accordingly, the present study was conceived as an exploratory team study intended to identify potential patterns of association and generate hypotheses for subsequent investigation rather than provide confirmatory population-level evidence. Second, the relatively large number of correlation analyses performed in relation to the sample size increases the risk of type I error. To address this issue, FDR correction was applied as a sensitivity analysis, and only two associations within the mechanical and metabolic load domain remained statistically significant after multiple-comparison control. Associations observed exclusively at the unadjusted level should therefore be interpreted cautiously and considered hypothesis-generating. Furthermore, the limited sample size necessarily results in wide confidence intervals and substantial uncertainty around correlation estimates, including those with large observed coefficients. Therefore, the magnitude of individual correlations should not be interpreted as providing precise estimates of the underlying population relationships. The use of a median split to form asymmetry groups, while facilitating exploratory group comparisons, is a methodological simplification that may obscure more nuanced dose–response relationships. The cross-sectional design precludes causal inference regarding the direction of the associations observed. Additionally, all participants were assessed first on the non-affected limb and subsequently on the affected limb. Although this approach was adopted to facilitate familiarisation and maximise the validity of maximal efforts, it may have introduced learning or fatigue effects that could have influenced the magnitude of the asymmetry values. Future research should examine the potential influence of limb-testing order in athletes with unilateral neurological impairments. Furthermore, a 10 s recovery interval was used between maximal contractions. Although this procedure was based on previous protocols and was applied consistently across participants, longer recovery periods may further reduce the potential influence of fatigue, particularly in athletes with neurological impairments. Therefore, the potential influence of incomplete recovery and fatigue-related effects between consecutive contractions cannot be completely excluded. Future studies should examine the influence of different recovery durations on maximal isometric force assessment in athletes with cerebral palsy. The present analyses were based on absolute force values expressed in Newtons. Although the sample was relatively homogeneous in terms of anthropometric characteristics, future studies may benefit from examining body-mass-normalised strength variables in addition to absolute force measures. Although three maximal contractions were performed for each condition, formal within-session reliability analyses were not conducted because the study was not designed as a reliability investigation. Future studies should determine the within-session reliability of belt-stabilised dynamometry specifically in elite CP football players.

Finally, match external load is influenced by sport class, playing position, tactical role, match status, and individual competitive exposure. Although external-load variables were normalised relative to individual playing time, the small and functionally heterogeneous sample precluded formal statistical adjustment or stratified analyses for these contextual factors. Consequently, the observed associations may partly reflect classification- or role-related differences rather than neuromuscular asymmetry alone. Moreover, several mechanical and metabolic load metrics are derived from shared locomotor and acceleration-related data and should not be considered statistically independent. Therefore, the similar associations observed across these variables may partly reflect shared underlying information. Future research should aim to replicate these exploratory findings in larger, multi-team samples and incorporate longitudinal and multivariable designs capable of accounting for sport class, playing position, match context, and other relevant player-level factors.

### 4.2. Practical Applications

The findings of this study have several implications for the assessment and monitoring of CP football players at the international level. First, inter-limb strength asymmetry, while substantially elevated in this population, should not be used as a standalone predictor of physical performance or as a basis for exclusion from high-intensity training. Practitioners are encouraged to interpret asymmetry indices in the context of the individual player’s impairment profile, sport class, and compensatory motor strategies, rather than applying symmetry thresholds derived from non-impaired athletic populations.

Second, the exploratory pattern observed between plantar flexor asymmetry and mechanical and metabolic match-load metrics identifies plantar flexor function as a potentially relevant variable for further investigation in players with spastic hemiparesis. However, given the interdependence of these external-load metrics and the potential influence of contextual and player-related factors, the present findings do not support interpreting plantar flexor asymmetry as an independent determinant of match physical demands. Belt-stabilised isometric dynamometry represents a portable and accessible method for neuromuscular profiling in field settings, and plantar flexor assessment may provide complementary information when interpreted alongside sport class, positional role, and match-load characteristics.

Third, the exploratory association between greater plantar flexor asymmetry and faster MAT-B performance should not be interpreted as evidence of a performance-enhancing effect of asymmetry. Rather, this unexpected pattern highlights the complexity of interpreting inter-limb asymmetry in athletes with unilateral neurological impairments and may reflect individual compensatory motor strategies. Given that this association did not remain significant after FDR correction, further research is required before specific practical recommendations can be derived.

Finally, the pronounced hamstring strength asymmetries observed (mean > 80%) underscore the need for ongoing monitoring of injury-risk-related neuromuscular variables in CP football players. Although the injury implications of such deficits require dedicated longitudinal investigation, practitioners should incorporate strength-focused training for the affected limb where functionally appropriate, while continuing to leverage the compensatory capacity of the non-affected limb to sustain match performance.

Although inter-limb asymmetry may represent a structural characteristic rather than a fully modifiable attribute in athletes with spastic hemiparesis, practitioners should routinely monitor unilateral strength profiles as part of the neuromuscular assessment process. Individualised interventions, including unilateral strength training, balance and coordination exercises, and appropriately prescribed plyometric training, may help optimise neuromuscular function and enhance sport-specific performance [30,31]. The primary objective should not necessarily be to eliminate inter-limb asymmetry, but rather to maximise functional capacity while considering each athlete’s individual impairment profile and movement strategies.

## 5. Conclusions

This study examined the associations between lower-limb isometric strength, inter-limb asymmetry, physical performance, and match external-load variables in international CP football players with spastic hemiparesis. Three key findings emerged. First, inter-limb strength asymmetry did not meaningfully differentiate physical performance outcomes across any of the field-based tests assessed, including sprint, change-of-direction, dribbling, and intermittent endurance. This supports the interpretation that asymmetry in this population functions as a structural characteristic of the neurological condition rather than a primary determinant of athletic capacity.

## Figures and Tables

**Table 1:** Lower-limb isometric strength and inter-limb asymmetry in players with spastic hemiparesis.

Muscle Group	Affected Limb (N)	Non-Affected Limb (N)	Asymmetry (%)	W (*p*)
Plantar flexors	79.29 ± 27.42	115.44 ± 51.11	46.58 ± 42.47	<0.01 (<0.001)
Adductors	123.38 ± 54.47	173.60 ± 57.08	49.83 ± 39.17	<0.01 (<0.001)
Hamstrings	131.88 ± 56.27	225.75 ± 66.21	81.81 ± 40.30	<0.01 (<0.001)

Values are presented as mean ± standard deviation. Strength values correspond to isometric force (Newtons) measured in the affected and non-affected limbs. *p*-values correspond to paired comparisons between affected and non-affected limbs using the Wilcoxon signed-rank test.

**Table 2:** Spearman’s correlations between lower-limb strength, inter-limb asymmetry, and physical performance variables.

Variable	Plantar Flexors (A)	Plantar Flexors (NA)	Adductors (A)	Adductors (NA)	Hamstrings (A)	Hamstrings (NA)	Plantar Flexor Asymmetry (%)	Adductors Asymmetry (%)	Hamstrings Asymmetry (%)
5 m sprint (s)	−0.47	−0.49	−0.39	−0.32	−0.19	0.04	−0.27	0.23	0.35
15 m sprint (s)	−0.32	−0.42	−0.26	−0.31	−0.08	−0.06	−0.29	0.10	0.11
30 m sprint (s)	−0.33	−0.52	−0.17	−0.32	0.02	−0.05	−0.47	−0.02	0.04
MAT (s)	−0.52	−0.46	−0.42	−0.42	−0.26	−0.06	−0.41	0.16	0.19
MAT-B (s)	−0.33	−0.58	−0.08	−0.49	−0.03	−0.21	−0.64 *	−0.33	−0.03
Dribbling ability (s)	−0.08	−0.28	0.06	−0.34	−0.01	−0.25	−0.39	−0.40	−0.09
Yo-Yo IR1 (m)	0.38	0.38	0.57	0.63 *	0.51	0.53	0.11	−0.09	−0.27

Values represent Spearman’s correlation coefficients (ρ). * *p* < 0.05. A: affected limb; NA: non-affected limb.

**Table 3:** Spearman’s correlations between inter-limb asymmetry and match external-load variables grouped by functional domains.

Variable	Plantar Flexor Asymmetry (%)	Adductors Asymmetry (%)	Hamstrings Asymmetry (%)
**Global locomotor load**			
Total distance per min (m·min^−1^)	−0.14	−0.05	0.04
Average velocity (km·h^−1^)	−0.43	−0.05	0.17
Locomotor intensity distribution			
Low-intensity distance (m·min^−1^)	0.02	0.26	0.38
Moderate-intensity distance (m·min^−1^)	−0.40	−0.16	−0.03
High-intensity distance (m·min^−1^)	0.28	−0.24	−0.24
**Neuromuscular actions (accelerations/decelerations)**			
Accelerations per min (n·min^−1^)	0.54	0.30	0.19
Decelerations per min (n·min^−1^)	0.54	0.30	0.19
Max acceleration (m·s^−2^)	0.46	−0.13	−0.14
Max deceleration (m·s^−2^)	−0.44	−0.06	0.04
Avg acceleration (m·s^−2^)	−0.48	−0.05	0.03
Avg deceleration (m·s^−2^)	0.31	0.02	0.11
High-intensity accelerations (n·min^−1^)	0.16	−0.17	−0.29
High-intensity decelerations (n·min^−1^)	0.15	−0.19	−0.27
Distance in high-intensity accelerations (m·min^−1^)	0.55	−0.08	−0.22
Distance in high-intensity decelerations (m·min^−1^)	0.49	−0.04	−0.21
Distance in moderate accelerations (m·min^−1^)	−0.25	−0.01	0.03
Distance in moderate decelerations (m·min^−1^)	−0.18	−0.09	−0.08
**Explosive and sprint actions**			
Explosive distance (m·min^−1^)	0.06	0.02	−0.01
Sprints per min (n·min^−1^)	0.39	−0.10	−0.20
Sprint distance per min (m·min^−1^)	0.44	−0.16	−0.28
Maximum velocity (km·h^−1^)	0.54	−0.08	−0.13
**Mechanical and metabolic load**			
Player load per min (AU·min^−1^)	−0.62 *	0.15	0.27
Mechanical work per min (kJ·min^−1^)	−0.84 **	−0.26	−0.12
Metabolic power (W·kg^−1^)	−0.83 **	−0.27	−0.09
High metabolic load distance (m·min^−1^)	−0.24	−0.18	−0.10

* *p* < 0.05; ** *p* < 0.01. Variables are grouped according to functional domains of external load: global locomotor load, locomotor intensity distribution, neuromuscular actions, explosive/sprint actions, mechanical and metabolic load.

**Table 4:** Differences in physical performance between higher- and lower-asymmetry groups.

**Physical Performance Variable**	**Plantar Flexor Asymmetry**				
	**Higher**	**Lower**	**U**	***p***	**r**
5 m sprint (s)	1.18 ± 0.06	1.20 ± 0.09	16.00	0.927	0.07
15 m sprint (s)	2.74 ± 0.08	2.84 ± 0.24	16.50	0.854	0.10
30 m sprint (s)	4.75 ± 0.13	4.89 ± 0.36	19.00	0.537	0.27
MAT (s)	5.76 ± 0.20	6.02 ± 0.41	20.00	0.429	0.33
MAT-B (s)	8.43 ± 0.42	8.89 ± 0.97	22.00	0.247	0.47
Dribbling ability (s)	2.67 ± 0.56	2.87 ± 0.78	19.00	0.537	0.27
Yo-Yo IR1 (m)	1088 ± 314	1227 ± 500	17.00	0.783	0.13
**Physical Performance Variable**	**Adductor Asymmetry**				
	**Higher**	**Lower**	**U**	***p***	**r**
5 m sprint (s)	1.18 ± 0.06	1.20 ± 0.09	13.00	0.783	−0.13
15 m sprint (s)	2.73 ± 0.09	2.85 ± 0.23	16.50	0.854	0.10
30 m sprint (s)	4.76 ± 0.14	4.88 ± 0.35	17.00	0.792	0.13
MAT (s)	5.83 ± 0.21	5.96 ± 0.44	14.00	0.931	−0.07
MAT-B (s)	8.59 ± 0.47	8.76 ± 1.01	18.00	0.662	0.20
Dribbling ability (s)	2.71 ± 0.55	2.84 ± 0.80	20.00	0.429	0.33
Yo-Yo IR1 (m)	1127 ± 336	1200 ± 505	13.00	0.783	−0.13
**Physical Performance Variable**	**Hamstrings Asymmetry**				
	**Higher**	**Lower**	**U**	***p***	**r**
5 m sprint (s)	1.17 ± 0.05	1.21 ± 0.10	13.00	0.783	−0.13
15 m sprint (s)	2.72 ± 0.09	2.86 ± 0.23	16.50	0.854	0.10
30 m sprint (s)	4.75 ± 0.15	4.90 ± 0.35	17.00	0.792	0.13
MAT (s)	5.80 ± 0.22	6.00 ± 0.43	14.00	0.931	−0.07
MAT-B (s)	8.50 ± 0.48	8.85 ± 0.98	18.00	0.662	0.20
Dribbling ability (s)	2.69 ± 0.56	2.86 ± 0.79	20.00	0.429	0.33
Yo-Yo IR1 (m)	1100 ± 330	1215 ± 510	13.00	0.783	−0.13

Values correspond to Mann–Whitney U test results comparing higher- vs. lower-asymmetry groups. Effect size is reported as rank-biserial correlation (r). No statistically significant differences were observed between groups (*p* > 0.05).

## Data Availability

Data are contained within the article.

## Abbreviations

CP	Cerebral palsy
COD	Change of direction
MVIC	Maximal voluntary isometric contraction
MAT	Modified Agility Test
MAT-B	Modified Agility Test with ball
Yo-Yo IR1	Yo-Yo Intermittent Recovery Test Level 1
IFCPF	International Federation of Cerebral Palsy Football
IMU	Inertial measurement unit
FT1	Football class 1
FT2	Football class 2
FT3	Football class 3
ICC	Intraclass correlation coefficient
SD	Standard deviation
ρ	Spearman’s rank correlation coefficient
GPS	Global Positioning System

## References

[1] Rosenbaum P., Paneth N., Leviton A., Goldstein M., Bax M., Damiano D., Dan B., Jacobsson B. (2007). A report: The definition and classification of cerebral palsy April 2006. Dev. Med. Child Neurol. Suppl..

[2] Graham H.K., Rosenbaum P., Paneth N., Dan B., Lin J.P., Damiano D.L., Becher J.G., Gaebler-Spira D., Colver A., Reddihough D.S. (2016). Cerebral palsy. Nat. Rev. Dis. Primers.

[3] Clewes O., Skerritt C., Kumar R., Day M., Bhatt H., Sherrat F., Sharma S., Lindley R. (2024). A systematic review of the neuromuscular impairments of spastic cerebral palsy and the evidence base for interventions. J. Pediatr. Rehabil. Med..

[4] Yanci J., Castillo D., Iturricastillo A., Urbán T., Reina R. (2018). External match loads of footballers with cerebral palsy: A comparison among sport classes. Int. J. Sports Physiol. Perform..

[5] Bishop C., Turner A., Read P. (2018). Effects of inter-limb asymmetries on physical and sports performance: A systematic review. J. Sports Sci..

[6] Fox K.T., Pearson L.T., Hicks K.M. (2023). The effect of lower inter-limb asymmetries on athletic performance: A systematic review and meta-analysis. PLoS ONE.

[7] Helme M., Tee J., Emmonds S., Low C. (2021). Does lower-limb asymmetry increase injury risk in sport? A systematic review. Phys. Ther. Sport.

[8] Buchheit M., Simpson B.M. (2017). Player-Tracking Technology: Half-Full or Half-Empty Glass?. Int. J. Sports Physiol. Perform..

[9] Bradley P.S., Ade J.D. (2018). Are Current Physical Match Performance Metrics in Elite Soccer Fit for Purpose or Is the Adoption of an Integrated Approach Needed?. Int. J. Sports Physiol. Perform..

[10] Andersson C., Grooten W., Hellsten M., Kaping K., Mattsson E. (2003). Adults with cerebral palsy: Walking ability after progressive strength training. Dev. Med. Child Neurol..

[11] Roldan A., Henríquez M., Iturricastillo A., Castillo D., Yanci J., Reina R. (2022). To what degree does limb spasticity affect motor performance in para-footballers with cerebral palsy?. Front. Physiol..

[12] Maggiolo J.F., Henríquez M., Moya-Ramón M., Peña-González I. (2025). Impact of inter-limb anthropometric asymmetries on physical performance in international footballers with spastic hemiplegia. Apunt. Sports Med..

[13] de Almeida M.B., Oliveira C., Ornelas G., Soares T., Souto J., Póvoa A.R., Ferreira L.M.A., Ricci-Vitor A.L. (2023). Intra-Rater and Inter-Rater Reliability of the Kinvent Hand-Held Dynamometer in Young Adults. Med. Sci. Forum..

[14] Taylor N.F., Dodd K.J., Graham H.K. (2004). Test-retest reliability of hand-held dynamometric strength testing in young people with cerebral palsy. Arch. Phys. Med. Rehabil..

[15] Verschuren O., Ketelaar M., Takken T., Van Brussel M., Helders P.J., Gorter J.W. (2008). Reliability of hand-held dynamometry and functional strength tests for the lower extremity in children with cerebral palsy. Disabil. Rehabil..

[16] Stark T., Walker B., Phillips J.K., Fejer R., Beck R. (2011). Hand-held dynamometry correlation with the gold standard isokinetic dynamometry: A systematic review. PM&R.

[17] Buckinx F., Croisier J.L., Reginster J.Y., Dardenne N., Beaudart C., Slomian J., Leonard S., Bruyère O. (2017). Reliability of muscle strength measures obtained with a hand-held dynamometer in an elderly population. Clin. Physiol. Funct. Imaging.

[18] Bishop C., Read P., Chavda S., Turner A. (2019). Asymmetries of the lower limb: The calculation conundrum in strength training and conditioning. Strength Cond. J..

[19] Gómez-Carmona C.D., Bastida-Castillo A., García-Rubio J., Ibáñez S.J., Pino-Ortega J. (2019). Static and dynamic reliability of WIMU PRO™ accelerometers according to anatomical placement. Proc. Inst. Mech. Eng. Part P J. Sports Eng. Technol..

[20] Exell T.A., Irwin G., Gittoes M.J.R., Kerwin D.G. (2012). Implications of intra-limb variability on asymmetry analyses. J. Sports Sci..

[21] Parkinson A.O., Apps C.L., Morris J.G., Dodd J., Lewis M.G.C. (2021). The calculation, thresholds and reporting of inter-limb strength asymmetry: A systematic review. J. Sports Sci. Med..

[22] Madruga-Parera M., Bishop C., Fort-Vanmeerhaeghe A., Beltran-Valls M.R., Skok O.G., Romero-Rodríguez D. (2020). Interlimb asymmetries in youth tennis players: Relationships with performance. J. Strength Cond. Res..

[23] Henríquez M., Sadarangani K.P., Cornejo M.I., Peña-González I., Yanci J., Reina R. (2025). Interlimb asymmetries in football players with coordination impairments: Implications for classification and training. Int. J. Sports Physiol. Perform..

[24] Sarabia J.M., Roldan A., Henríquez M., Reina R. (2021). Using decision trees to support classifiers’ decision-making about activity limitation of cerebral palsy footballers. Int. J. Environ. Res. Public Health.

[25] Runciman P., Derman W., Ferreira S., Albertus-Kajee Y., Tucker R. (2015). A descriptive comparison of sprint cycling performance and neuromuscular characteristics in able-bodied athletes and Paralympic athletes with cerebral palsy. Am. J. Phys. Med. Rehabil..

[26] Dos’Santos T., Thomas C., Jones P.A., Comfort P. (2018). Asymmetries in isometric force-time characteristics are not detrimental to change of direction speed. J. Strength Cond. Res..

[27] Impellizzeri F.M., Marcora S.M., Coutts A.J. (2019). Internal and external training load: 15 years on. Int. J. Sports Physiol. Perform..

[28] Croisier J.L., Ganteaume S., Binet J., Genty M., Ferret J.M. (2008). Strength imbalances and prevention of hamstring injury in professional soccer players: A prospective study. Am. J. Sports Med..

[29] Guan Y., Bredin S.S.D., Taunton J., Jiang Q., Wu N., Warburton D.E.R. (2022). Association between inter-limb asymmetries in lower-limb functional performance and sport injury: A systematic review of prospective cohort studies. J. Clin. Med..

[30] Papale O., Festino E., De Maio M., Di Rocco F., Zema S., Cortis C., Fusco A. (2026). Acute Effects of a Mini-Trampoline Training Session for Improving Normalized Symmetry Index in Participants with Higher Baseline Inter-Limb Asymmetry. Healthcare.

[31] Sammoud S., Negra Y., Bouguezzi R., Granacher U., Chaabene H. (2024). Effects of plyometric jump training on measures of physical fitness and lower-limb asymmetries in prepubertal male soccer players: A randomized controlled trial. BMC Sports Sci. Med. Rehabil..

